# Consistency and reproducibility of the VMAT plan delivery using three independent validation methods

**DOI:** 10.1120/jacmp.v12i1.3373

**Published:** 2010-11-16

**Authors:** Varatharaj Chandraraj, Sotirios Stathakis, Ravikumar Manickam, Carlos Esquivel, Sanjay S Supe, Nikos Papanikolaou

**Affiliations:** ^1^ Departments of Radiation Oncology and CTRC The University of Texas Health Science Center San Antonio TX USA; ^2^ Department of Radiation Physics Kidwai Memorial Institute of Oncology Bangalore India

**Keywords:** VMAT, quality assurance, DAVID, DynaLog files, PTW seven29 ARRAY

## Abstract

The complexity of VMAT delivery requires new methods and potentially new tools for the commissioning of these systems. It appears that great consideration is needed for quality assurance (QA) of these treatments since there are limited devices that are dedicated to the QA of rotational delivery. In this present study, we have evaluated the consistency and reproducibility of one prostate and one lung VMAT plans for 31 consecutive days using three different approaches: 1) MLC DynaLog files, 2) *in vivo* measurements using the multiwire ionization chamber DAVID, and 3) using PTWseven29 2D ARRAY with the OCTAVIUS phantom at our Varian Clinac linear accelerator. Overall, the three methods of testing the reproducibility and consistency of the VMAT delivery were in agreement with each other. All methods showed minimal daily deviations that contributed to clinically insignificant dose variations from day to day. Based on our results, we conclude that the VMAT delivery using a Varian 2100CD linear accelerator equipped with 120 MLC is highly reproducible.

PACS numbers: 87.55.Qr and 87.56.Fc

## I. INTRODUCTION

Intensity‐modulated radiotherapy (IMRT) treatment is capable of producing complex dose distributions that can conform to even a concave target volume.^(^ ^1^ ^)^ IMRT has been established as an accurate, reliable and efficient method of delivering conformal radiotherapy^(^ ^2^ ^,^ ^3^ ^)^ maximizing the tumor dose and minimizing the dose to normal tissue. Newer techniques such as intensity‐modulated arc therapy (IMAT) were first introduced by Yu^(^ ^4^ ^)^ and consisted of modulation of multiple arcs to achieve appropriate levels of dose target conformity and critical organ sparing. Another approach developed by Otto^(^ ^5^ ^)^ involved an aperture‐based optimization method called volumetric‐modulated arc therapy (VMAT), which typically requires one gantry rotation and produces dose distributions equivalent to IMRT. VMAT is able to deliver radiation in a single 360° arc which may produce more conformal dose distributions when compared to other methods such as SS or SW IMRT. VMAT is five‐to‐fifteen times more efficient, in terms of treatment time and reduction in monitor units, as compared to the helical or serial tomotherapy.^(^ ^5^ ^)^

The high flexibility of VMAT delivery and the complexity of the systems involved will require new methods and potentially new tools for the commissioning of these systems. It appears that great consideration is needed for quality assurance (QA) of these treatments since there are limited devices that are dedicated to the QA of rotational delivery. QA of IMRT delivery techniques is critical to ensure accurate delivery of optimized treatment plans. A combination of pretreatment dosimetric plan verification with increased mechanical and dosimetric constancy checks is typically needed.^(^ ^6^ ^)^ The dosimetric verification of treatment plans using *in vivo* dosimetry in IMRT includes TLD detectors^(^ ^7^ ^)^ and MOSFET detectors.^(^ ^8^ ^,^ ^9^ ^)^ Several researchers have reported on the patient‐specific QA of VMAT using various devices (ionization chamber arrays, diode detector arrays, etc).^(^ ^10^ ^–^ ^12^ ^)^ However, the reliability of *in vivo* point measurements obtained in a high gradient region produced by IMRT is subject to question.^(^ ^13^ ^)^ A new *in vivo* commercial device, DAVID (device for advance verification of IMRT deliveries) developed by PTW (PTW, Freiburg, Germany), consists of a flat multiwire ionization chamber to be placed in the accessory holder of the treatment head, with the ability of on‐line recording of the beam profile. The signal from each detection wire is associated with the opening width of one leaf pair of the MLC, and the chamber is constructed from translucent materials to minimize interference with the light localizing system of the treatment head.

The proper implementation of VMAT, using dynamic multileaf collimation (DMLC) techniques, requires a thorough understanding of leaf motion during delivery. The effects of leaf motion can be studied using film dosimetry, an electronic portal imaging device and the ionization measurements.^(^ ^14^ ^,^ ^15^ ^)^ These techniques provide dosimetric information but do not provide detailed information for diagnosing delivery problems. More specific evaluation of the control system and MLC function can be done using the information contained in the dynamic log files, or DynaLog files in the case of Varian MLCs.^(^ ^16^ ^)^ These files contain leaf position and dose fraction information recorded every 50 milliseconds. This information can be used as part of the overall system QA to evaluate the function of different parts of an IMRT system.^(^ ^17^ ^)^

In this present study, we have evaluated the consistency and reproducibility of a prostate and a lung VMAT plan case for 31 consecutive days using three different approaches: 1) MLC DynaLog files, 2) *in vivo* measurements using the multiwire ionization chamber DAVID, and 3) using PTW seven29 ARRAY with OCTAVIUS (PTW, Freiburg, Germany) phantom. All measurements were done on our Varian Clinac (Varian Medical Systems, Palo Alto, CA) linear accelerator with a 120 Leaf MLCs.

## II. MATERIALS AND METHODS

Two full arc VMAT plans of a prostate and a lung treatment were chosen for this study. Constant dose rate and gantry speed settings were used during the optimization and delivery of these plans. The volume of the prostate PTV to be treated was 236.7 cc and the prescription was 180 cGy per fraction, while the prescription for the lung target was 212 cGy with a volume of 50.2 cc. The monitor units for these two plans were 399 and 909 for the prostate and lung plans, respectively.

All plans were delivered in VMAT mode using a Varian 2100CD linear accelerator capable of VMAT delivery. The linac is equipped with120 Millennium multileaf collimator. The PTW seven29 and OCTAVIUS phantom were set up at the isocenter, and the PTW DAVID was inserted in the wedge tray slot of the linac. For each plan, a pretreatment delivery was performed and the measurements obtained (DynaLog files, planar dose with seven29, and the fluence from DAVID) were used as reference measurements. The remaining 30(n=30) consecutive daily measurements were compared against the reference.

### A. DynaLog file description

A DynaLog file is a file record of the actual dose fraction (dose dynamic) versus actual MLC leaf positions from the either dynamic treatment or a segmental treatment, recorded in ASCII format. A dynamic treatment is a treatment during which the MLC leaves, collimator or gantry moves while the beam is on, and both the dose rate and the speed of the leaves are continuously adjusted by the control system. The DynaLog data are taken every 50 ms by the MLC controller. The record continues until the dynamic treatment is completed or terminated. This record may be written to a file (DynlogA.txt and DynlogB.txt) on the control system computer after each IMAT plan delivery. (A complete file description may be found in the Varian 2003 User Manual.) These files can be transferred to another computer for detailed analysis of the operation of the DMLC function after each IMAT plan is delivered. Due to the fact that the dose rate and the gantry speed were kept constant, dose fraction information can be extracted from the DynaLog files.

The analysis of these DynaLog files have been carried out by in‐house programming in MATLAB (The MathWorks, Natick, MA) by converting these ASCII files in to a 552×552 matrix that can be visualized as a gray level fluence image of the corresponding QA plan. Further analysis was carried out in two ways: first, by subtracting the 552×552 matrix of the daily measurement from the reference measurement and then calculating the mean, standard, maximum and minimum values of the differences; second, by using the fluence images that were saved in Tagged Image File Format (TIFF) and were subsequently analyzed using the Radiation Imaging Technology (RIT113, Radiological Imaging Technology, Colorado Springs, CO) radiation therapy QA software. The quantitative analysis between the reference and a daily fluence distribution was evaluated using DTA and gamma index. The tolerance of 1% intensity difference and 1 mm DTA and gamma tolerance ≤1 was set for the analysis, since relaxed criteria (i.e., 3% and 3 mm) show no differences during comparison of consecutive deliveries.

### B. PTW DAVID description

*In vivo* verification of IMRT photon fluence distribution using a transmission radiation detector positioned at the head of the machine is a novel QA technique for IMRT. In this work, we used the DAVID system, which is able to perform such quality assurance measurement while the patient is treated.^(^ ^18^ ^)^ Each measurement wire monitors the opening of a leaf pair. The measured dose length product is correlated to the opening of the leaf pair and the supplied dose. The evaluation software compares the dose measured during radiotherapy to a reference dose, which was taken for each leaf pair during a reference measurement. DAVID can be used independently of the IMRT method (step & shoot, sliding window or dynamic arc).

The DAVID unit for Varian MLC 120 is a transparent, segmented, multiwire ionization chamber with 80 wires. It is designed for operation in the Varian LINAC of 60 leaf pairs; 40 interior leaf pairs are covered by one wire and the 20 exterior leaf pairs (10 on each side) are each covered by two measuring wires.

**Figure 1:**
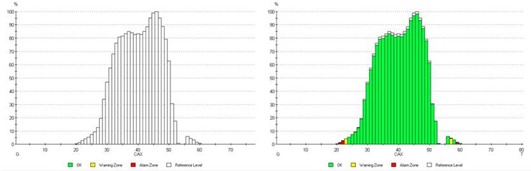
Reference measurement with DAVID is shown as a transparent bar (a) and measurement graph of a single fraction (b).

Measurement with DAVID consists of two parts: one is reference measurements and the other for each session measurement. Before daily measurements can be obtained, a reference measurement must be acquired. A reference measurement can either be performed on a phantom during plan verification or during the first fraction. During each delivery, the fluence delivered to the patient is compared with one of the reference measurement. The deviation of the measured values from the reference values should be within predefined limits. The progress of the measurement can be watched by means of the measurement progress bar, the measurement graphic and the display of the results. In measurement graphic, the measured value bars will be colored according to the adjusted warning and alarm levels. The display of results will show the maximum deviation of a measurement channel, the mean deviation of all measurement channels, and the deviation of the total dose from the reference measurement, as shown in Fig. 1. In the present study, above 5% of the maximum deviation was considered to be the alarm level (presented as red color bars), between 3% to 5% were considered warning levels (yellow color bars) and less than 3% were considered as passing.

### C. PTW seven29 ARRAY with OCTAVIUS phantom description

The third method of measuring consistency and reproducibly of VMAT QA plan is by using the OCTAVIUS phantom with the seven29 ion chamber array consisting of 729 air‐filled chambers. The OCTAVIUS phantom is made up of polystyrene (density of $1.04\;g/{\text{cm}}^{3}$) and the dimensions of this octagon shaped phantom are 32.0 cm in diameter and 32.0 cm in length. In the seven29 array, the ionization chambers are equally spaced, 1 cm center to center, and the maximum detector area covered by the chamber array is $27\times 27\;{\text{cm}}^{2}$. A buildup layer surrounds each of the vented ionization chambers and is made of PMMA. Each chamber has a size of $0.5\;\times 0.5\times 0.5\;{\text{cm}}^{3}$. The linear dimensions of the 2D array seven29 are $2.2\;\times 30.0\times 42.0\;{\text{cm}}^{3}$. The maximum and minimum measurement interval for scanning the ion chamber matrix and processing the acquired data were 999 ms and 400 ms, respectively. The device allows us to measure absorbed dose to water (Gy) and absorbed dose rate to water ($\text{Gy}\;\min^{-1}$) in continuous operation mode. The 2D array is calibrated for absolute dosimetry in a ${\;}^{60}\text{Co}$ photon beam at the PTW secondary standard dosimetry laboratory. An on‐site factor correcting for the quality of the beam was calculated and supplied to the detector acquisition software prior to measurement. A correction factor was obtained before each measurement session. In the present study, the quantitative analysis between the first day measurement which is taken as reference and the consecutive measurements were evaluated using gamma difference and absolute dose difference. The tolerance of 3% dose difference, 3 mm DTA and gamma tolerance of ≤1 was set for the analysis, according to the AAPM TG119 report recommendations.^(^ ^19^ ^)^ Moreover, profile and isodose distributions were used for comparison along with absolute dose differences.

## III. RESULTS

**Figure 2:**
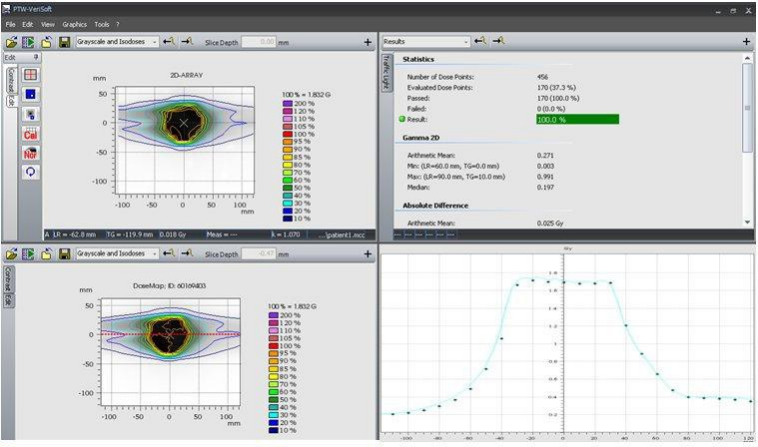
Comparison between the measurement and TPS calculated isodose and profile for a prostate plan.

The patient‐specific QA results for the two plans in our study were evaluated first. The planar doses calculated from the treatment planning system were compared to the measurements and the evaluation was based on the gamma index, isodose profile comparison and profile comparison. Both plans had a passing gamma index of 100% when 3 mm and 3% dose difference were selected as the evaluation criteria. The results as evaluated by the VeriSoft 4.0 software (PTW, Freiburg, Germany) for the prostate plan are shown in Fig. 2. These measurements were used as the reference for the daily evaluation of the delivery.

### A. DynaLog file analysis

**Figure 3:**
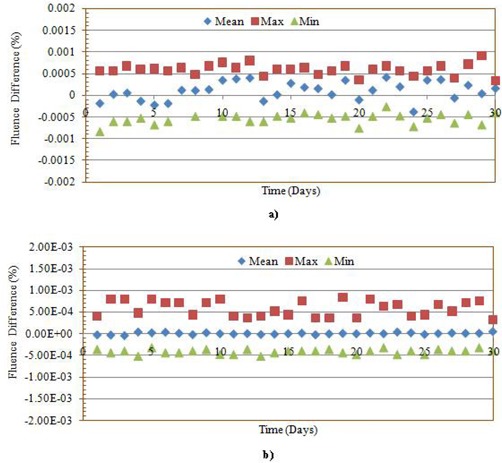
The deviation of the fluence matrix of 30‐days of measurement data versus reference for: a) prostate, and b) lung VMAT QA plan.

**Figure 4:**
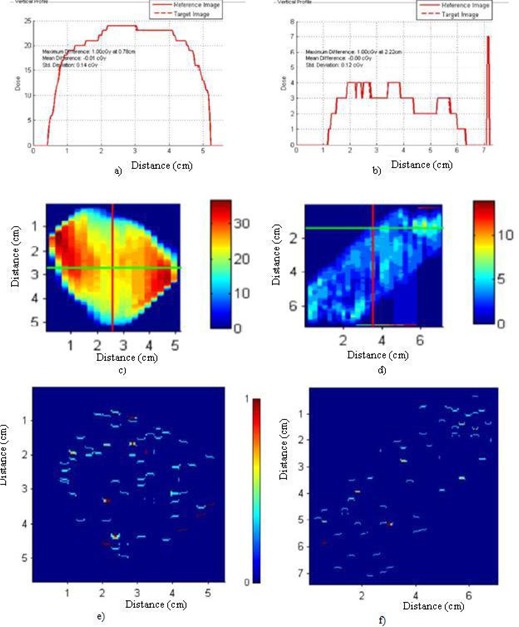
The comparison of the vertical profiles for one of the session versus the reference measurement data for: a) prostate and b) lung VMAT plan using RIT analysis; the corresponding fluence image is shown in c) and d) for the prostate and the lung case, respectively. Gamma comparison for one of the sessions versus the reference measurement data for: e) prostate and f) lung VMAT plan using RIT analysis by taking the gamma criteria as 1% and 1 mm.

All the DynaLog files of the two VMAT QA plans were converted to corresponding 552×552 size fluence matrix using an in‐house MATLAB program. For analysis, the reference matrix was subtracted from the consecutive day matrices using our MATLAB program. The maximum, minimum and mean deviation of this fluence matrix are shown in Figs. 3(a) and 3(b) for a prostate and a lung case, respectively. Since the values of the difference between the two matrices were very low, we have analyzed the DynaLog files by converting each fluence matrix into TIFF images. The RIT113 v5.2 radiation therapy QA software was employed to compare the reference against each of the consecutive measurements. This analysis showed that the variations between the reference fluence and the daily fluence were very low (see Fig. 4). The mean gamma index (1% and 1 mm DTA) was as low as 0.01±0.08. Results of the remaining 30 comparisons corresponding to the rest of the deliveries are not shown but have very similar outcomes to those plotted in Fig. 4.

### B. PTW DAVID analysis

**Figure 5:**
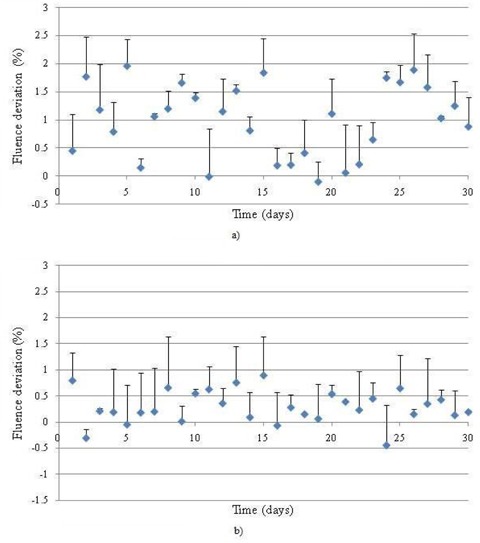
Mean fluence deviation of the measured values for 30 days versus the reference measurement using PTW DAVID for: a) prostate and b) lung.

In the analysis of PTW DAVID, we can identify which leaf pairs are responsible for the recorded deviation. In the software display, the daily measurement is overlaid and deviation levels are displayed in a color scheme. In our 30‐day measurement period, the maximum variations were within 3% for both VMAT QA plans and the percentage mean deviation, as shown in Fig. 5 for prostate and lung cases, respectively. The error bars displayed in the figure represent the maximum deviation calculated by the DAVID software.

### C. PTW seven29 array analysis

**Figure 6:**
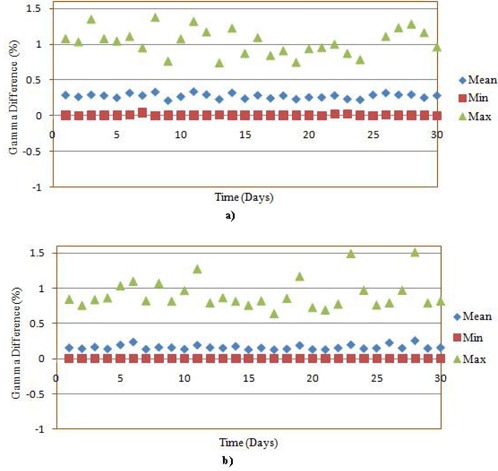
Gamma difference (%) between the 30‐days measured values versus the reference measurement using PTW seven29 ARRAY for: a) prostate and b) lung.

**Figure 7:**
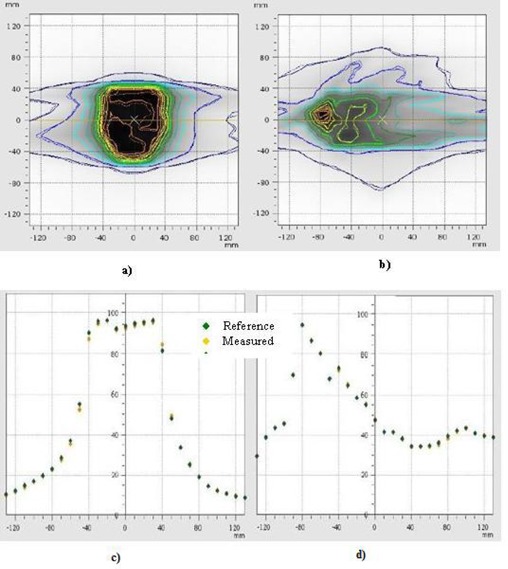
Comparison of isodose curves between reference versus one of the thirty‐day measurements for: a) prostate and b) lung cases using PTW seven29 ARRAY. The corresponding profile comparisons are shown in c) and d).

In the PTW seven29 array detector analysis, the percentage of gamma difference between the daily measured fluence and the reference fluence for the prostate and lung cases are shown in Fig. 6. The isodose and profile comparison of the reference measurement and one of the 30‐day sessions for both cases are in agreement, as shown in Fig. 7. The differences between the consecutive days of treatment are found to be very small and the gamma index passing rate was, on average, above 99.5% in both cases. The maximum gamma index values observed in the prostate case were about 1.5, while for the lung case it was about 1.4.

## IV. DISCUSSION

The reproducibility of VMAT delivery was verified in the present study with the aid of three independent methods. The daily dose fluence was verified using the DAVID system, the delivered dose was measured using the seven29 ionization chamber array, and the MLC‐dose delivery was confirmed by the analysis of the daily DynaLog files as they were recorded during each delivery.

The daily fluence analysis using PTW DAVID showed minimal variations (Fig. 6) with the mean being at maximum 2% (on average 1.06%±0.7% for prostate and 0.32%±0.2% for lung). It was observed from the results that the mean deviation is higher for the prostate case. The reason for this may be attributed to the fact that the complexity of the prostate plan is greater than the lung plan. In the lung plan, the leaves form an almost circular aperture and the leaves move during gantry rotation in an attempt to maintain the aperture. In the prostate case, there are a larger number of leaves that travel greater path lengths in order to create the necessary modulation. That was simply due to the fact that the prostate target volume was considerably larger with more surrounding structures (bladder, rectum and femoral heads) that are needed to be spared than with the PTV for the lung case.

Similar results were observed in the analysis of the delivered dose as recorded by the seven29 ionization chamber array. The mean gamma was lower in the lung case than in the prostate case. This is supported by the fact that the lung target was smaller (50.2 cc) and hence the ratio of the chambers measuring signal to the total number of chambers was smaller. Therefore, more ionization chambers in the array with zero signals detected are contributing to the gamma index and increasing the gamma index passing rate. In cases like this, it is recommended that a threshold signal value is used (signal above 10%) and only detectors with signal above the threshold are used in the analysis.^(^ ^19^ ^)^ The recorded MLC locations and MU delivered through each segment obtained by the DynaLog files show minimal differences. Most of the differences observed were within 1 mm between the measured and calculated position of the leaves.

Overall, the three methods of testing the reproducibility and consistency of the VMAT delivery were in agreement with each other. Minimal differences were observed through the analysis of the DynaLog files and the DAVID. It should be noted that part of the differences in dose measured with the seven29 can be attributed to setup inaccuracies rather than variations in the daily delivery. Such setup uncertainties can be in the order of 1 mm in all directions.

## V. CONCLUSIONS

In this study, the reproducibility and consistency of VMAT delivery were examined. DynaLog files, *in vivo* measurements of the delivered fluence and planar dose measurements were employed to assess the VMAT delivery for 30 consecutive daily treatments. All methods showed minimal daily deviations that contributed to clinically insignificant dose variations from day to day. Based on our results, we conclude that the VMAT delivery using a Varian 2100CD linear accelerator equipped with 120MLC is highly reproducible.

## ACKNOWLEDGEMENTS

This work has been supported by a UICC International Cancer Technology Transfer Fellowship, and with Federal funds from the National Cancer Institute, National Institutes of Health under Contract NO2‐CO‐41101.
